# Quantifying the shift in social contact patterns in response to non-pharmaceutical interventions

**DOI:** 10.1186/s13362-020-00096-y

**Published:** 2020-12-01

**Authors:** Zachary McCarthy, Yanyu Xiao, Francesca Scarabel, Biao Tang, Nicola Luigi Bragazzi, Kyeongah Nah, Jane M. Heffernan, Ali Asgary, V. Kumar Murty, Nicholas H. Ogden, Jianhong Wu

**Affiliations:** 1 Fields-CQAM Laboratory of Mathematics for Public Health (MfPH), York University, Toronto, Ontario Canada; 2 Laboratory for Industrial and Applied Mathematics, York University, Toronto, Ontario Canada; 3 Department of Mathematical Sciences, University of Cincinnati, Cincinnati, OH USA; 4 CDLab—Computational Dynamics Laboratory, Department of Mathematics, Computer Science and Physics, University of Udine, 33100 Udine, Italy; 5 Modelling Infection and Immunity Lab, Centre for Disease Modelling, Department of Mathematics and Statistics, York University, Toronto, Ontario Canada; 6 Disaster & Emergency Management, School of Administrative Studies & Advanced Disaster & Emergency Rapid-Response Simulation (ADERSIM), York University, Toronto, Ontario Canada; 7 Department of Mathematics, University of Toronto, Toronto, Ontario Canada; 8 The Fields Institute for Research in Mathematical Sciences, Toronto, Ontario Canada; 9 Public Health Risk Sciences Division, National Microbiology Laboratory, Public Health Agency of Canada, St-Hyacinthe, Quebec Canada

**Keywords:** COVID-19, Intervention evaluation, Mathematical modelling, Transmission model, Heterogeneous mixing, Non-pharmaceutical interventions

## Abstract

Social contact mixing plays a critical role in influencing the transmission routes of infectious diseases. Moreover, quantifying social contact mixing patterns and their variations in a rapidly evolving pandemic intervened by changing public health measures is key for retroactive evaluation and proactive assessment of the effectiveness of different age- and setting-specific interventions. Contact mixing patterns have been used to inform COVID-19 pandemic public health decision-making; but a rigorously justified methodology to identify setting-specific contact mixing patterns and their variations in a rapidly developing pandemic, which can be informed by readily available data, is in great demand and has not yet been established. Here we fill in this critical gap by developing and utilizing a novel methodology, integrating social contact patterns derived from empirical data with a disease transmission model, that enables the usage of age-stratified incidence data to infer age-specific susceptibility, daily contact mixing patterns in workplace, household, school and community settings; and transmission acquired in these settings under different physical distancing measures. We demonstrated the utility of this methodology by performing an analysis of the COVID-19 epidemic in Ontario, Canada. We quantified the age- and setting (household, workplace, community, and school)-specific mixing patterns and their evolution during the escalation of public health interventions in Ontario, Canada. We estimated a reduction in the average individual contact rate from 12.27 to 6.58 contacts per day, with an increase in household contacts, following the implementation of control measures. We also estimated increasing trends by age in both the susceptibility to infection by SARS-CoV-2 and the proportion of symptomatic individuals diagnosed. Inferring the age- and setting-specific social contact mixing and key age-stratified epidemiological parameters, in the presence of evolving control measures, is critical to inform decision- and policy-making for the current COVID-19 pandemic.

## Introduction

In response to the current COVID-19 pandemic, interventions aimed at controlling local transmission such as school and non-essential business closures, physical distancing, contact tracing, enhanced surveillance and diagnostic testing have been adopted throughout many nations of the world [1]. The efficacy of these measures and their influence on the trajectory of local epidemics has been quantified in a series of mechanistic modelling studies [2–5], as well as in systematic reviews and meta-analyses [6–8]. Additionally, key factors associated with demographic heterogeneities such as age-dependent social contact mixing and susceptibility to infection and their implications on transmission patterns of COVID-19 have been explored in prior works [9–11]. While there has been increasing utilization of age- and setting-specific contact mixing patterns to inform COVID-19 pandemic public health decision-making, rigorously quantifying their variations during a pandemic intervened by changing public health measures presents a significant challenge in the absence of time and resources to conduct a high-quality contact survey (e.g., as in prior work [11–13]). Moreover, a methodology for identifying such age- and setting (workplace, household, school and community)-specific contact mixing patterns using readily available data is in great demand and has yet to be established. Such contact mixing patterns are key for the retroactive evaluation and proactive assessment of the effectiveness of different age- and setting-specific interventions. Further, a comprehensive modelling approach that integrates key heterogeneities by age/setting and a generalized intervention package accounting for evolving non-pharmaceutical interventions, diagnostic testing, contact tracing, and case isolation may be utilized for a broad spectrum of risk assessment, preparedness planning, reopening measures, scenario analysis and intervention evaluation.

Understanding age- and setting (workplace, household, school and community)-specific transmission is fundamental for retrospectively assessing the effects of non-pharmaceutical interventions on transmission, and essential for planning (smart) relaxation of measures while protecting the most vulnerable populations. The interruption of contact routes (through interventions such as physical distancing, the closing of schools, workplaces and community gathering places) naturally shifts contact patterns among different settings. We may thus expect to observe an increase in transmission in some settings. Understanding these changes is critical to avoid unwanted increases in transmission amongst vulnerable portions of the population. Since many of the non-pharmaceutical intervention measures taken to counteract the spread of COVID-19 are unprecedented and highly disruptive to personal life, their effects are not completely understood and largely depend on the adherence of individuals and their behavior. Hence, retrospectively assessing the consequences of interventions provides important tools to evaluate the effectiveness of such measures, and to prospectively inform the expected outcomes of relaxations [11, 14] and possible reintroduction of intervention measures in the event of resurgence.

In addition to contact mixing patterns, recent attention has been given to age-specific epidemiological and clinical parameters for COVID-19 [9, 10, 15], as they allow one to assess which portions of the population may be key drivers of the epidemic or which portion may be most vulnerable to infection. For instance, children and adolescents are likely key contributors to the spread of respiratory infections, as they tend to mix assortatively and have relatively high contact rates [16]. Consequently, school closure is one of the first non-pharmaceutical interventions considered to mitigate the spread of an emerging respiratory infection. However, if children and adolescents were demonstrated to have low transmissibility and/or susceptibility, their contribution to infection could be minor despite higher contact rates and highly assortative mixing patterns. In this light, understanding the age-specific contribution to infection in terms of transmissibility and susceptibility is key for planning interventions [9] and designing effective vaccination strategies and other pharmaceutical interventions.

Here we propose a general methodology to investigate the age- and setting-specific contribution to the transmission of an infectious disease and illustrate the approach by taking the COVID-19 epidemic in Ontario, Canada as a case. We develop and utilize a suitable transformation accounting for the specific demographic profile in Ontario so that for a given choice of age group divisions, age-specific contact patterns can be constructed from seminal works on contact mixing [16, 17]. The transmission model we propose accounts for two key control measures for communicable diseases, diagnosis of cases as a result of symptoms, and isolation through contact tracing [2–5]. By fitting the model output to age-stratified incidence data, we inform critical parameters including the age-stratified susceptibility to infection. Finally, by incorporating information about the features and timing of non-pharmaceutical interventions and the consequent shifts in contact patterns, the fitting procedure allows us to quantify such changes and retrospectively inform the effect of intervention measures on the social contact patterns and the setting of transmission events. Specifically, in Ontario we consider four key periods of control measure escalation which we denote by distinct phases: phase 0, monitoring and international travel advisories (until March 13); phase 1, public school closure (March 14–17); phase 2, state of emergency declaration and physical distancing advisories (March 18–23); phase 3, closure of non-essential workplaces (March 24–May 16). We explore the robustness of our estimates using several layers of uncertainty analysis.

## Methods

### Transmission model

We extended the COVID-19 transmission dynamics model introduced in prior studies [2–5] to include age structure. The model captures essential epidemic features and key public health interventions. The population is divided into susceptible (*S*), exposed (*E*), asymptomatic infectious (*A*), infectious with symptoms (*I*), and recovered (*R*) compartments according to the epidemiological status of individuals, and further into diagnosed and isolated (*D*), quarantined susceptible ($S_{q}$), and isolated exposed ($E_{q}$) compartments based on control interventions involving testing, contact tracing, quarantine and isolation. In particular, the model accounts for contact tracing, where a proportion, *q*, of individuals exposed to the virus are quarantined. The quarantined individuals can either move to the compartment $E_{q}$ or $S_{q}$, depending on whether they are effectively infected or not, while the other proportion, $1 - q$, consists of individuals exposed to the virus who are missed from contact tracing and, therefore, move to the exposed compartment *E* once effectively infected, or stay in the compartment *S* otherwise. Furthermore, the population is stratified into *n* age groups, where the social interactions between age groups are described via a contact matrix, *C*, which incorporates information about age-specific contacts in different settings, including households, schools, workplaces and the general community. We assumed that the susceptibility and diagnosis rates depend on the specific age group, whereas all remaining parameters are constant across age groups.

**Figure 1:**
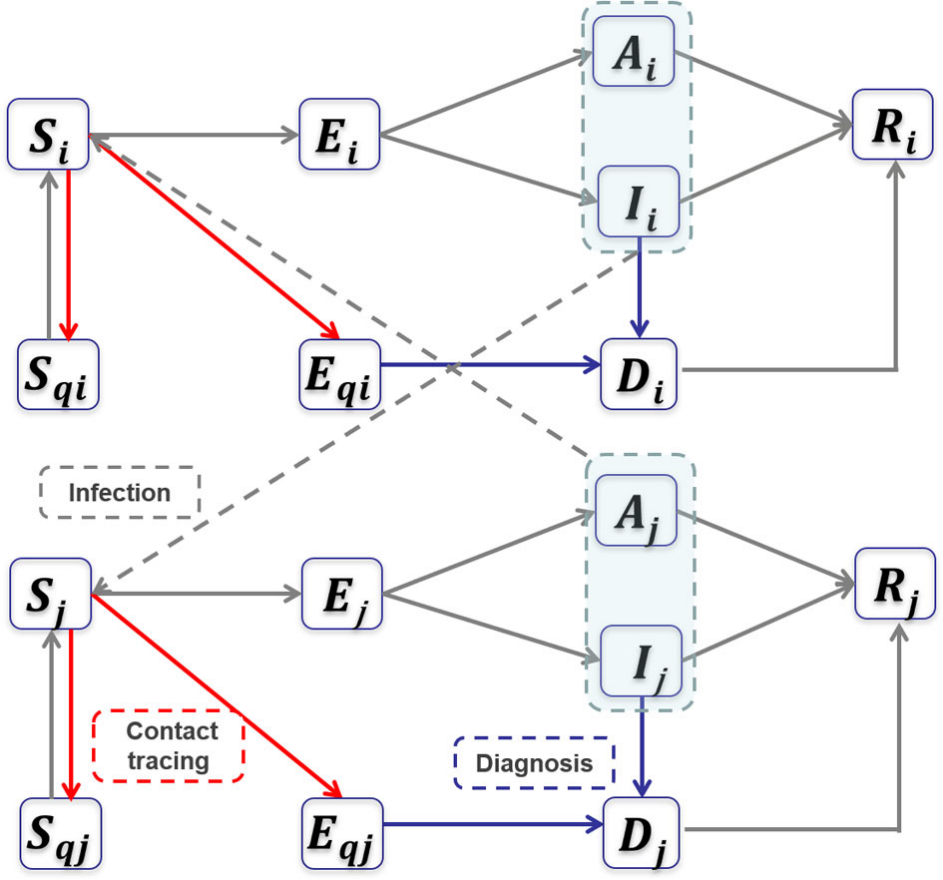
Flowchart of the transmission model. Schematic diagram of the transmission model accounting for a generalized package of control measures. For the construction of the mathematical model, see Methods

The model reads

1 $$ \begin{gathered} S'_{i} = - \sum _{j=1}^{n} \bigl( \beta _{i} C_{ij} +q (1- \beta _{i} ) C_{ij} \bigr) S_{i} ( I_{j} + \theta A_{j} )/ N_{j} + \lambda S_{qi}, \\ E'_{i} = \sum_{j=1}^{n} \beta _{i} C_{ij} (1-q )S_{i} ( I_{j} + \theta A_{j} )/ N_{j} -\sigma E_{i}, \\ I'_{i} = \sigma \varrho E_{i} - ( \delta _{Ii} + \gamma _{I} ) I_{i}, \\ A'_{i} = \sigma ( 1 - \varrho ) E_{i} - \gamma _{A} A_{i}, \\ S'_{qi} = \sum_{j=1}^{n} (1- \beta _{i} ) C_{ij} q S_{i} ( I_{j} + \theta A_{j} )/ N_{j} -\lambda S_{qi}, \\ E'_{qi} = \sum_{j=1}^{n} \beta _{i} C_{ij} q S_{i} ( I_{j} + \theta A_{j} )/ N_{j} - \delta _{q} E_{qi}, \\ D'_{i} = \delta _{Ii} I_{i} + \delta _{q} E_{qi} - ( \alpha + \gamma _{D} ) D_{i}, \\ R'_{i} = \gamma _{I} I_{i} + \gamma _{A} A_{i} + \gamma _{D} D_{i} \end{gathered} $$

for each age group $i=1,\ldots,n$, where $N_{i}$ ($N_{j} $) denotes the total population in age group *i* (*j*). Additionally, we allowed several parameters to be time-dependent during phase 3, to account for a gradual adaptation of the society to the stricter physical distancing measures. The transmission dynamics in an age-stratified population is illustrated in Fig. 1 with all model compartments and parameters defined in Tables 1 and 2, respectively. The model parameter definitions are also provided in the subsequent section.

**Table 1:** List of compartments in the transmission model for COVID-19 in Ontario, Canada

Model variable	Description
$S_{i}$	Susceptible population
$E_{i}$	Exposed population
$I_{i}$	Symptomatic infected population
$A_{i}$	Asymptomatic infected population
$S_{q_{i}}$	Quarantined susceptible population
$E_{q_{i}}$	Quarantined exposed population
$D_{i}$	Diagnosed population
$R_{i}$	Recovered population

The index *i* refers to the age groups *i* = 1,2,3,4,5,6. Hence, there are six model variables for each stage, one for each age class *i*.

**Table 2:** List of parameters used in the transmission model for COVID-19 in Ontario, Canada

	Definition	Mean (Std)	Source
Parameter			
$p_{1}^{H}$	Relative increase of the weight of the contact matrix for household settings from phase 0 to phase 1	0.1320 (0.0866)	Estimated
$p_{1}^{C}$	Relative increase of the weight of the contact matrix for community settings from phase 0 to phase 1	0.0685 (0.0642)	Estimated
$p_{2}^{H}$	Relative increase of the weight of the contact matrix for households from phase 1 to phase 2	0.2832 (0.1807)	Estimated
$p_{2}^{C}$	Relative decrease of the weight of the contact matrix for community settings from phase 1 to phase 2	0.3283 (0.1575)	Estimated
$p_{3}^{H}$	Final relative increase of the weight of the contact matrix for household settings in phase 3	0.0436 (0.0593)	Estimated
$p_{3}^{W}$	Final relative decrease of the weight of the contact matrix for workplace settings in phase 3	0.5921 (0.0820)	Estimated
$p_{3}^{C}$	Final relative decrease of the weight of the contact matrix for community settings in phase 3	0.7888 (0.1697)	Estimated
$r_{H}$	Exponential increase in household contact rate	0.0379 (0.0532)	Estimated
$r_{W}$	Exponential decrease in workplace contact rate	0.4711 (0.0853)	Estimated
$r_{C}$	Exponential decrease in community contact rate	0.1019 (0.0461)	Estimated
$\beta_{i}$	Probability of transmission per contact, age-dependent	Table 4	Estimated
$q_{0}$	Fraction of quarantined exposed individuals phase 0–2	0.1187 (0.0645)	Estimated
$q_{b}$	Maximum fraction of quarantined individuals exposed	0.7272 (0.0583)	Estimated
$r_{q}$	Exponential increase in quarantine fraction	0.0282 (0.0022)	Estimated
*σ*	Transition rate of exposed individuals to the infected class	1/5	[18]
*λ*	Rate at which the quarantined uninfected contacts were released into the wider community	1/14	[5]
*ϱ*	Probability of having symptoms among infected individuals	0.7036	[2]
$\delta _{Ii}$	Transition rate of symptomatic infected individuals to the quarantined infected class	Table 4	Estimated
$\delta _{q}$	Transition rate of quarantined exposed individuals to the quarantined infected class	0.3409 (0.1137)	Estimated
$\gamma _{I}$	Removal rate of symptomatic infected individuals	0.1957	[2]
$\gamma _{A}$	Removal rate of asymptomatic infected individuals	0.139	[5]
$\gamma _{D}$	Removal rate of quarantined diagnosed individuals	0.2	[19]
*α*	Disease-induced death rate	0.008	[19]
*θ*	Modification factor of asymptomatic infectiousness	0.0275	[2]
Initial values			
$S_{i} (0)$	Initial susceptible population	Table 4	Data [20]
$E_{i} (0)$	Initial exposed population	Table 4	Estimated
$I_{i} (0)$	Initial symptomatic infected population	Table 4	Estimated
$A_{i} (0)$	Initial asymptomatic infected population	Table 4	Estimated
$S_{q_{i}}(0)$	Initial quarantined susceptible population	0	Assumed
$E_{q_{i}}(0)$	Initial quarantined exposed population	0	Assumed
$D_{i} (0)$	Initial diagnosed population	Table 4	Incidence data
$R_{i} (0)$	Initial recovered population	0	Assumed

For the estimated parameters, we report the mean and standard deviation of the fitting results of the 1000 bootstrap realizations. For non-fitted parameters, the source is reported.

#### Parameter definitions

The susceptibility to infection $\beta _{i}$ (i.e., the probability of a susceptible individual being infected upon contact with an infectious individual) and the diagnosis rate from the symptomatic compartment $\delta _{Ii}$ were assumed to be age-specific. We assumed the age-dependent susceptibility to infection and the diagnosis rates to be constant for each age class during phases 0–3.

The incubation period $1/\sigma _{i} =1/\sigma $, the quarantine period $1/\lambda _{i} =1/\lambda $, the modification factor for asymptomatic transmission $\theta _{ij} =\theta $, the recovery rates $\gamma _{Ai} = \gamma _{A}$, $\gamma _{Di} = \gamma _{D}$, $\gamma _{Ii} = \gamma _{I}$, the disease-induced death rate $\alpha _{i} =\alpha $, the ratio of symptomatic infections $\varrho _{i} =\varrho $, and the quarantine proportion and diagnosis rates, $q_{i} =q$ and $\delta _{qi} = \delta _{q}$, were assumed to be equal for all age groups. All age-independent parameters are listed in Table 2. Most parameters were assumed to be constant through all the escalation phases, except for the quarantine rate, which was assumed to be exponentially increasing in phase 3, in order to capture the intensification of contact tracing effort from the public health system. We set

$$ q ( t ) = \textstyle\begin{cases} q_{0}, &t< T_{2}, ( \text{phase 0--2} ), \\ ( q_{0} - q_{b} ) e^{- r_{q} ( t- T_{2} )} + q_{b}, &t\geq T_{2}, ( \text{phase 3} ), \end{cases} $$

where $q_{0}$ is the constant quarantine proportion prior to March 24, $q_{b}$ is the maximum quarantine proportion after March 24 and $r_{q}$ represents the exponential rate of increase.

In addition, we assumed time-dependent contact rates in phase 3, to capture the gradual adaption of physical distancing in various locations during this period. To capture the change in contact patterns during different phases of escalation of physical distancing, we defined the social contact matrix *C* piecewise as follows:

$$ C ( t ) = \textstyle\begin{cases} C^{0},& T_{S} < t< T_{0}, ( \text{phase 0} ),\\ C^{1}, &T_{0} < t< T_{1}, ( \text{phase 1} ),\\ C^{2}, &T_{1} < t< T_{2}, ( \text{phase 2} ),\\ C^{3} ( t ), &T_{2} < t< T, ( \text{phase 3} ), \end{cases} $$

where $T_{S}$, $T_{0}$, $T_{1} $, $T_{2} $ and *T* mark as the start date of phase 0, 1, 2, 3 and the end date of phase 3 (before the de-escalation phases). The contact matrix in phase 3 was assumed to be time-dependent, to describe a gradual adaptation of the society to adhere to the stricter measures enforced.

Each matrix $C^{0}$, $C^{1}$, $C^{2}$, $C^{3} (t)$ was constructed as a linear combination of the setting-specific contact matrices. Specifically, let $C^{H}$, $C^{W}$, $C^{C}$, $C^{S}$ denote the baseline contact matrices quantifying the daily contact rate of physical and nonphysical contacts in household, workplace, community and school settings. The superscripts *H*, *W*, *C*, *S* associated with contact matrices and model parameters will be used to refer to household, workplace and community settings, respectively. The entries of the contact matrices are the number of daily social contacts of a single individual in age class *i* with individuals in age class *j* (units contacts/day as defined by those contacts believed to be relevant for the spread of respiratory illnesses) [16]. In what follows, $p_{l}^{k} >0$ denotes the relative increase (or decrease) in the weight of the daily individual contact rate matrix in setting *k* from intervention phase *l*. That is, the superscript *k* can be *H*, *W*, *C* or *S* to associate parameters with household, workplace, community or school settings, respectively. Note that, because of the different nature of contacts in different settings, a decrease in contact in one setting does not necessarily mean an equal increase in a different setting in terms of either weight or numbers (and vice versa). In fact, each escalation phase could change the contact patterns both qualitatively and quantitatively, and contacts lost in one setting do not necessarily shift completely to another. For this reason, we did not assume a specific relation between the coefficients $p_{l}^{k}$ in each phase, allowing contact patterns in each setting to change independently. In the following, the contact matrix in each escalation phase is defined and discussed individually.

#### Phase 0: monitoring and international travel advisories

We assumed the contact mixing in the absence of physical distancing and mandatory closures is the linear combination of the four setting-specific matrices, each with an equal weight of 1:

$$ C^{0} = C^{H} + C^{W} + C^{C} + C^{S}. $$

#### Phase 1: public school closure

The phase 1 mixing matrix is given by:

$$ C^{1} = \bigl( 1+ p_{1}^{H} \bigr) C^{H} + C^{W} + \bigl( 1+ p_{1}^{C} \bigr) C^{C} + 0 C^{S}. $$

Here $p_{1}^{H}$ is the percent increase in the weight of the household contact matrix from phase 0 to phase 1. Similarly, $p_{1}^{C}$ is the percent increase in the weight of the community contact matrix from phase 0 to phase 1. In the remaining equations, the school contact matrix is no longer written explicitly to simplify their appearance.

#### Phase 2: physical distancing advisories

The phase 2 mixing matrix is given by:

$$ C^{2} = \bigl( 1+ p_{2}^{H} \bigr) \bigl( 1+ p_{1}^{H} \bigr) C^{H} + C^{W} + \bigl( 1- p_{2}^{C} \bigr) \bigl( 1+ {p}_{1}^{C} \bigr) C^{C}. $$

Here $p_{2}^{H}$ is the percent increase in the weight of the household contact matrix from phase 1 to phase 2. Also, $p_{2}^{C}$ is the percent reduction in the weight of the community contact rate matrix from phase 1 to phase 2 due to physical distancing advisories and closures.

#### Phase 3: closure of non-essential workplaces

During phase 3, we allowed the coefficients of each setting-specific matrix to be time-dependent (either exponentially increasing or decreasing), to capture the gradual adaptation of society to the new more restrictive measures. Specifically, we assumed that workplace and community contacts were gradually decreasing, whereas household contact was gradually increasing, possibly with different rates. The phase 3 mixing matrix is given by:

$$\begin{aligned} \begin{aligned} C^{3} (t) = {}&\bigl[\bigl(1+ p_{3}^{H} \bigr)- e^{- r_{H} ( t- T_{2} )} p_{3}^{H} \bigr] \bigl(1+ p_{2}^{H} \bigr) \bigl(1+ p_{1}^{H} \bigr) C^{H} \\ & {} + \bigl[ p_{3}^{W} e^{- r_{W} (t- T_{2} )} +\bigl(1- p_{3}^{W} \bigr)\bigr] C^{W} \\ &{}+ \bigl[ p_{3}^{C} e^{- r_{C} ( t- T_{2} )} +\bigl(1- p_{3}^{C} \bigr)\bigr]\bigl(1- p_{2}^{C} \bigr) \bigl(1+ p_{1}^{C} \bigr) C^{C}, \end{aligned} \end{aligned}$$

where $p_{3}^{H}$ is the maximal percent increase in the weight of the household contact matrix from phase 2 to phase 3. $p_{3}^{W}$ and $p_{3}^{C}$ are the maximal percent reduction in the weight of the workplace and community contact matrix, respectively, from phase 2 to phase 3 resulting from the closure of non-essential workplaces.

#### Age group subdivision

We stratified the population of Ontario into $n=6$ age groups, comprised of ages 0–5, 6–13, 14–17, 18–24, 25–64, 65+, which broadly represent children (ages 0–5), elementary and middle school (ages 6–13), high school (ages 14–17), university (ages 18–24), workforce (ages 25–64) and seniors (ages 65+), and we ascribe the indices $i=1, 2,\ldots, 6$ to these classes (Table 3). We considered six age groups since most physical distancing measures taken in Ontario have been formulated and implemented corresponding to these six age-groups.

**Table 3:** Details of the age groups used in transmission model (1) for COVID-19 in Ontario, Canada

Age range (years)	0–5	6–13	14–17	18–24	25–64	65+
Age class index (*i*)	1	2	3	4	5	6
Age class population (${N}_{i}$)	877,614	1,245,978	641,784	1,411,604	7,879,605	2,509,962

The age class population ($N_{i}$) refers to the population counts in year 2019.

#### Initial conditions

The initial susceptible populations were fixed as the total population of each age class in Ontario, Canada which were obtained from Statistics Canada [20]. We considered the initial fitting date ($t=0$) to be February 26, which marks the date at which sustained case accumulation began in Ontario. Therefore, we supposed there were initially no recovered individuals, that is $R_{i} ( 0 ) =0$ for each age class *i*. The initial diagnosed populations were fixed as the numbers of cumulative cases for each class till February 26. Similarly, we set $S_{qi} ( 0 ) = E_{qi} ( 0 ) =0$ for each age class *i*. Since the confirmed cases for the three youngest age groups (ages 0–17) are zero for at least one week after February 26, we set $E_{i} ( 0 ) = A_{i} ( 0 ) = I_{i} ( 0 ) =0$ for $i=1,2,3$, while we estimated the conditions for $i=4, 5, 6$. All the initial conditions for model (1) are listed in Table 2 and Table 4.

**Table 4:** Age-specific model parameter estimates in Ontario, Canada (mean and standard deviation)

Parameter	Age class					
	0–5	6–13	14–17	18–24	25–64	65+
$\delta _{Ii}$	0.0866 (0.0619)	0.1212 (0.0587)	0.1137 (0.0611)	0.0486 (0.0274)	0.3460 (0.0087)	0.3981 (0.0841)
$\beta _{{i}}$	0.0185 (0.0055)	0.0164 (0.0039)	0.0248 (0.0062)	0.0916 (0.0031)	0.1393 (0.0059)	0.5023 (0.0249)
$E_{i}(0)$	0	0	0	6 (1.0852)	6 (0.9363)	6 (0.7158)
$A_{i}(0)$	0	0	0	6 (0.6351)	6 (0.6590)	5 (0.6838)
$I_{i}(0)$	0	0	0	3 (0.0313)	10 (0.5206)	10 (0.8171)
$D_{i}(0)$	0	0	0	1	5	1
$S_{i}(0)$	877,614	1,245,978	641,784	1,411,604	7,879,605	2,509,962

The initial conditions $D_{i}(0)$ and $S_{i}(0)$ are fixed (not estimated), as well as $E_{i}(0)$, $A_{i}(0)$ and $I_{i}(0)$ for age groups *i* = 1,2,3.

### Data

We used prior results and data to construct the baseline social contact matrices for Ontario, Canada. We retrieved the age-stratified population estimates for Ontario in year 2019 and Canada in year 2006 from Statistics Canada [20]. As part of the POLYMOD project, social contact surveys in eight European countries were conducted between May 2005 and September 2006 to quantify age-specific contact heterogeneity [16]. Country-specific data on household structures, labor-force participation rates, and school enrolment were utilized to project the European social contact data to contact rates representative of 152 countries, including Canada [17]. We utilized the projected setting (household, workplace, community and school)-specific contact matrices for Canada [17] in this study and adapted them to represent mixing in Ontario.

To proceed with model fitting, there are several additional data sources utilized within this study. While in this case study data specific to Ontario was utilized, we note this approach is based on a general methodology that can be applied broadly. First, we utilized the timeline of interventions taken by the government of Ontario, Canada. The escalation of physical distancing measures in Ontario consisted of three major steps: public school closure (from March 14), declaration of state of emergency, with closure of public venues and events and physical distancing advisories (from March 18), and closure of non-essential establishments (from March 24). On May 16, selected non-essential activities had resumed, marking the end of the non-essential establishment closure in Ontario. Second, we utilized the age-stratified cumulative confirmed positive tests in Ontario, Canada. We obtained this data of the age-specific cumulative cases of COVID-19 in Ontario from the Ontario Ministry of Health, which was made available to us through the Ontario COVID-19 Modeling Consensus Table. Third, the age-structured demographic data specific to Ontario is available publicly by Statistics Canada [20]. These main sources of data enabled the fitting of mathematical model and all subsequent analyses.

### Contact mixing matrices

**Figure 2:**
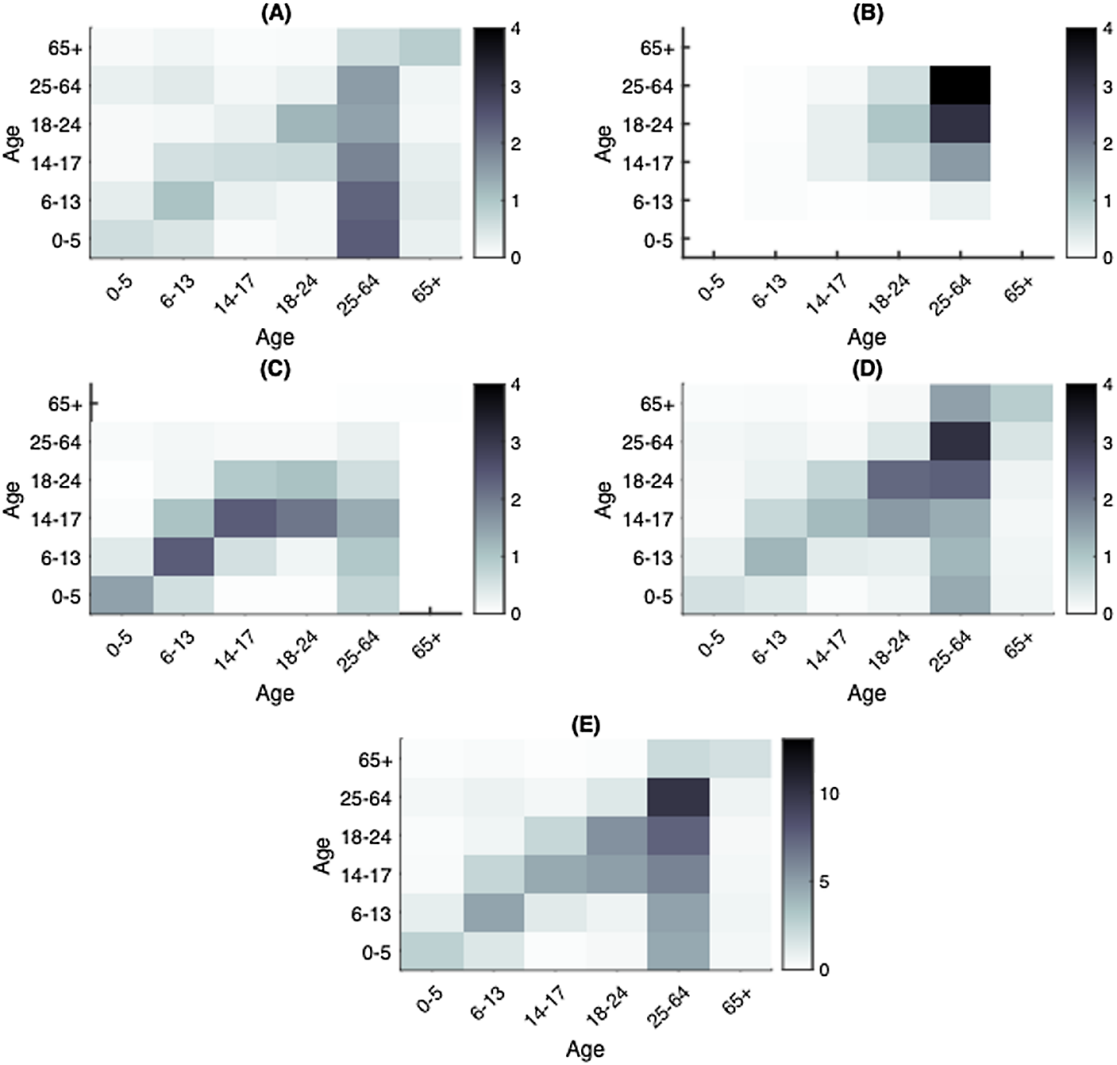
Heatmaps of estimated social contact matrices in Ontario, Canada. Age-specific contact mixing in the absence of physical distancing interventions in Ontario (**A**) Households, (**B**) Workplaces, (**C**) Schools, (**D**) Communities and other locations and (**E**) contact mixing in all four settings combined

We established the setting-specific contact matrices in the household, workplace, community, and school in Ontario, Canada denoted $C^{H}$, $C^{W}$, $C^{C}$, $C^{S}$, respectively (Fig. 2). We derived these mixing matrices from the Canada-specific contact matrices [17] through a series of transformations to account for the Ontario demographic profile and the desired age group division, then utilized these as baseline contact patterns in the absence of physical distancing measures. The details, including the specific definitions and mathematical formulation of the baseline contact matrices, are included in Appendix A: Baseline contact matrices.

### Model fitting procedure

To estimate model parameters, we fit the mathematical model to age-stratified cumulative incidence data. We first informed model (1) with several parameter values from existing studies (Table 2) and also the established social contact matrices. We then run model (1) forward from time $t = T_{s}$ (chosen as February 26, corresponding to the date at which sustained case accumulation began in Ontario) to time *T* (chosen as May 16, the date of first easing of restrictions), and determined parameters which minimize the least square error against the age-stratified cumulative incidence. In other words, we estimated parameters associated with model (1) by fitting to six lists of time series data representing cumulative incidence according to age class. To obtain confidence intervals for the estimated parameters, we used a bootstrap method to generate 1000 cumulative incidence time series from a Poisson distribution with mean given by the reported data and fitted the model to each dataset. We assumed a Poisson error structure in the newly reported cases to address noise in this time series data (for context of this topic, see [21]).

### The control reproduction number

The control reproduction number $R_{t}$ describes the average number of secondary cases that one random infected individual produces during its infectious period, under the control measures (diagnosis and quarantine). We obtained the control reproduction number of model (1) using the next generation method [22]. $R_{t}$ is the spectral radius of the next generation matrix $K(t)$. The (time-dependent) next generation matrix for the parameters considered in this paper (i.e. with $\beta _{i}$ and $\delta _{Ii}$ both age specific) is

$$ \bigl[K(t)\bigr]_{ij} = \bigl(1-q(t)\bigr) A_{j} \beta _{i} C_{ij} (t) N_{i} / N_{j} $$

for $i, j\in \{ 1, 2,\ldots, n \} $ where $A_{j} = \frac{\rho }{\delta _{I,j} + \gamma _{I}} + \frac{\theta ( 1-\rho )}{\gamma _{A}}$.

### Infectious contacts

We compared the estimated values for the mean infectious contact, i.e., the mean number of contacts per day that result in an infection with a homogeneous mixing model of similar scope [2]. In the age-structured model (1) each contact contributes differently to transmission; hence, the mean contact rates estimated from the age-structured model in each phase are not directly comparable with the contact rate obtained by fitting a homogeneous model, as done in previous work [2]. For the homogeneous model, this is computed as *βc*, where *c* and *β* denote the contact rate and probability of infection upon contact previously estimated, respectively [2]. For the age-structured model, we considered the combination $\frac{1}{N} \sum_{i} \beta _{i} N_{i} \sum_{j} C_{ij} (t)$, which accounts for the age-stratified susceptibility to infection.

### Contact rates and infections acquired in each setting

The setting-specific contact rate was computed based on each estimated setting-specific contact matrix and population profile in 2019 for Ontario. The mean connectivity, or the number of daily contacts averaged over all individuals in the population with mixing matrix *C*, is defined as

$$ \langle k\rangle = \frac{1}{N} \sum_{i,j} C_{ij} N_{i}. $$

The all-setting social contact rates were calculated from the sum of the setting-specific contact rates. For the details of the terminology, definitions, and methods associated with the contact mixing matrices, see Appendix: Baseline contact matrices.

We computed the infections acquired in each setting by using the estimated model parameters (Tables 2 and 4) and model (1). Specifically, the infections acquired in each setting were tracked in time as the sum of the inflow to the exposed (*E*) and exposed quarantined ($E_{q}$) compartments. We added four additional compartments to the mathematical model, one each for workplace, school, household and community setting, and using the estimated model parameters and setting-specific contact matrices, solve the system of ordinary differential equations to assess the infections acquired in each setting.

### Uncertainty analysis

The robustness of our estimates is explored with several layers of uncertainty analysis. First, we quantified parameters in terms of the uncertainty in reported cases by assuming a Poisson error structure and fit model (1) to 1000 corresponding realizations. The resulting uncertainty in the model fit is expressed in terms of uncertainty in the estimated model parameters. Second, we assessed the empirical distributions of several of the key estimated parameters. The empirical distributions of the age-specific susceptibility to infection and the estimated weights associated with the contact matrices were constructed to investigate the robustness of these estimates.

## Results

### Model fitting

**Figure 3:**
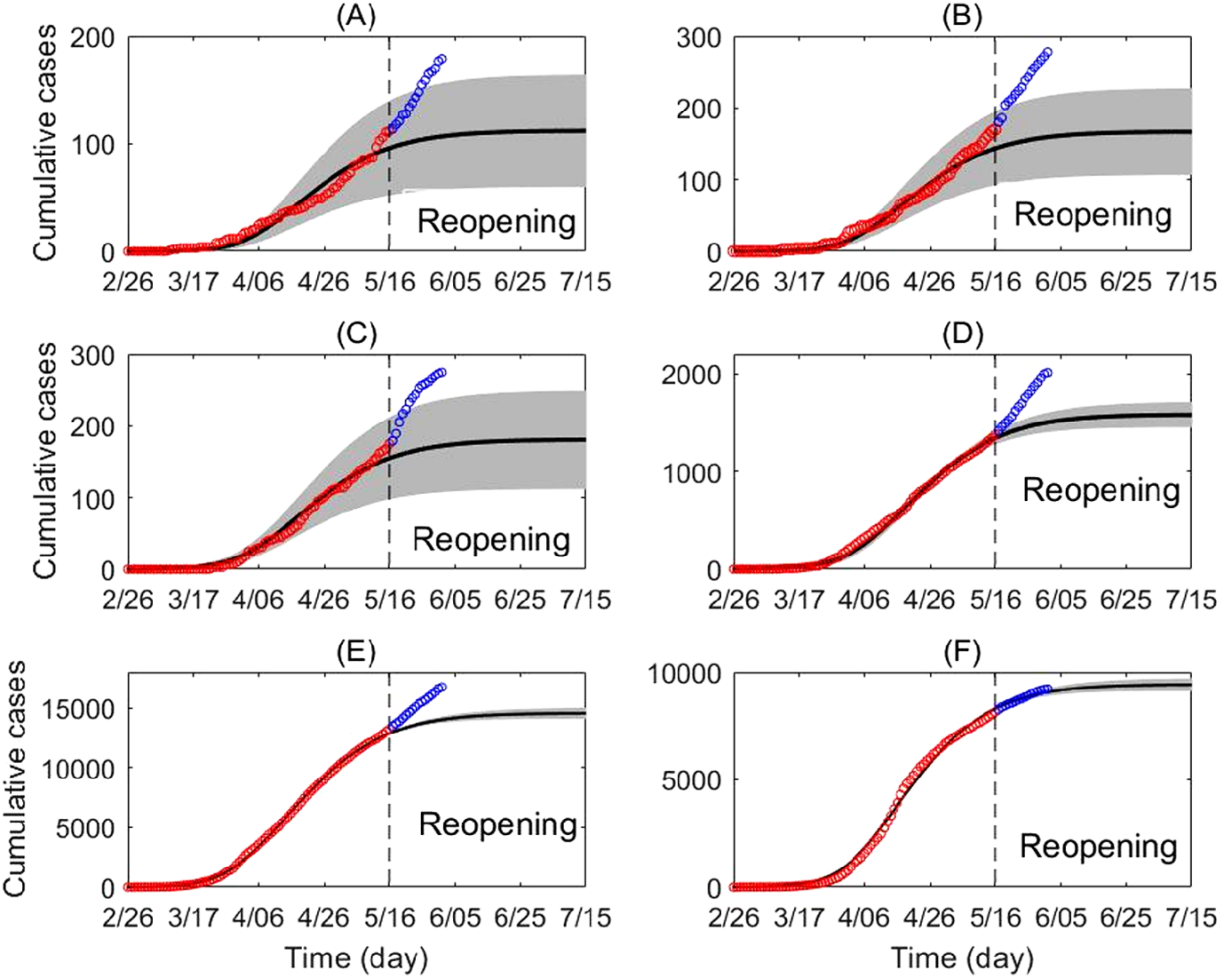
Cumulative incidence according to age class. Cumulative incidence according to age class (circles) and best fitting transmission model (line), with 95% confidence interval (gray region). The red circles represent data from February 26 to May 16 (fitted). The blue circles represent data May 17 to June 1 (not fitted). Cumulative incidence shown for (**A**) ages 0–5, (**B**) ages 6–13, (**C**) ages 14–17, (**D**) ages 18–24, (**E**) ages 25–64 and (**F**) ages 65+. For an explanation of the increase in reported cases after May 16, see Appendix B: Caution in interpretation

**Figure 4:**
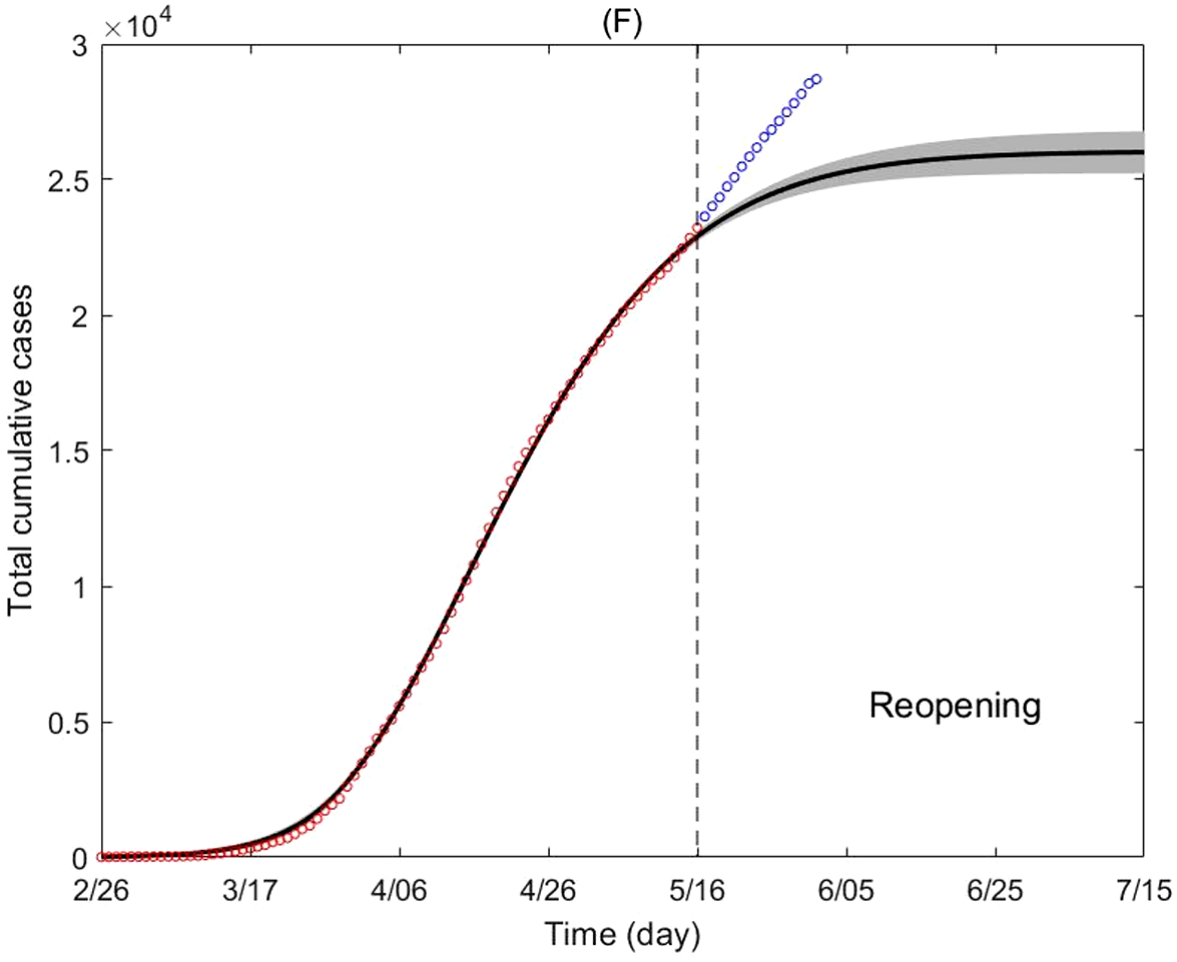
Cumulative incidence of all age classes combined. Cumulative incidence of all age classes (circles) and best fitting model (line), with 95% confidence interval (gray region). The red circles represent data from February 26 to May 16 (fitted). The blue circles represent data May 17 to June 1 (not fitted). For an explanation of the increase in reported cases after May 16, see Appendix B: Caution in interpretation

**Figure 5:**
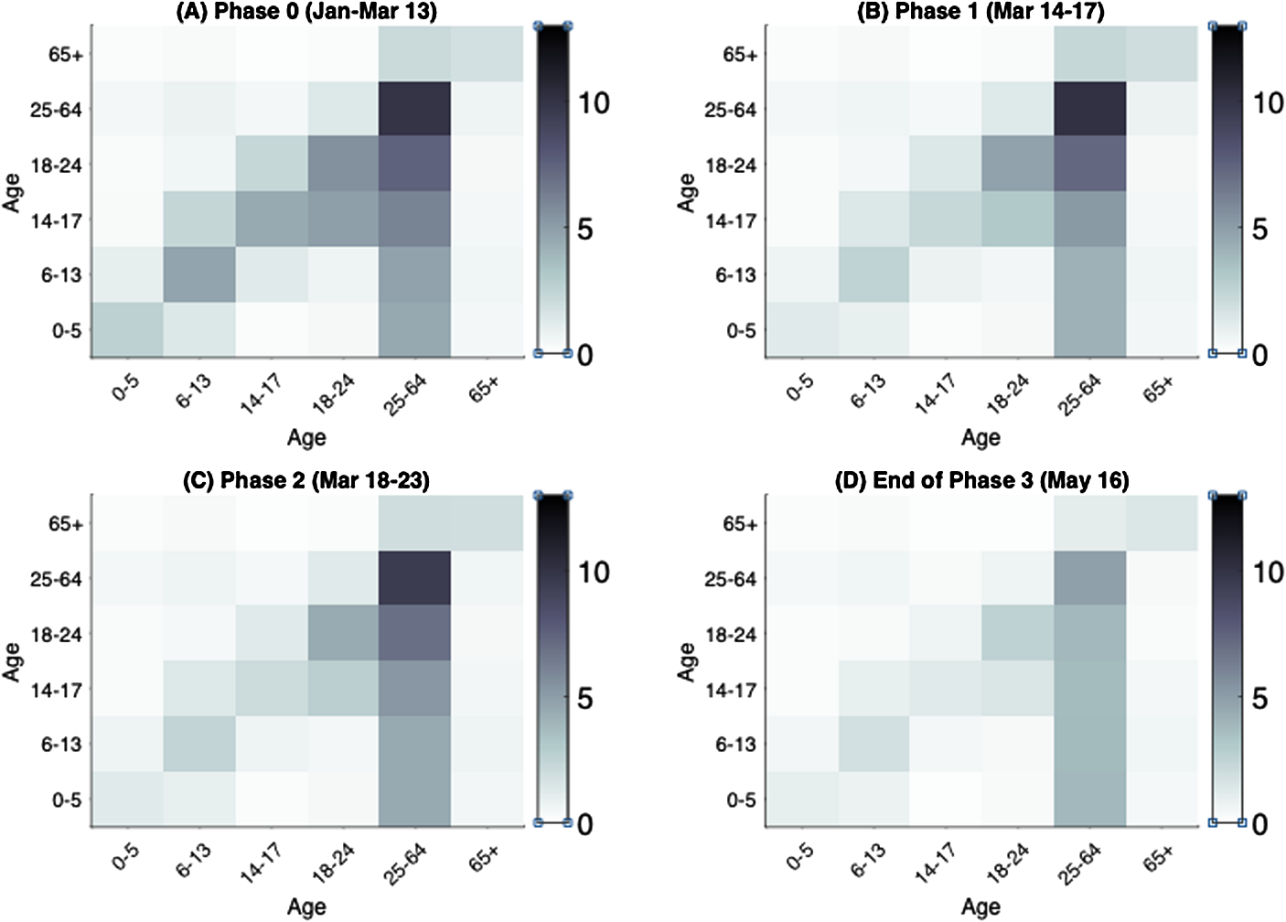
Age-specific contact mixing pattern estimated for each escalation phase. Shown are the heatmaps of contact matrices for all settings (workplace, school, community, and household) combined. The intensity of the color of an entry corresponds to the magnitude of the contact rate between the intersecting age classes. The row of the matrix represents the contactor age class and the column represents the age class of the contactee. Heatmaps depicted for contact mixing in (**A**) phase 0, (**B**) phase 1, (**C**) phase 2 and (**D**) the end of phase 3 on May 16

**Figure 6:**
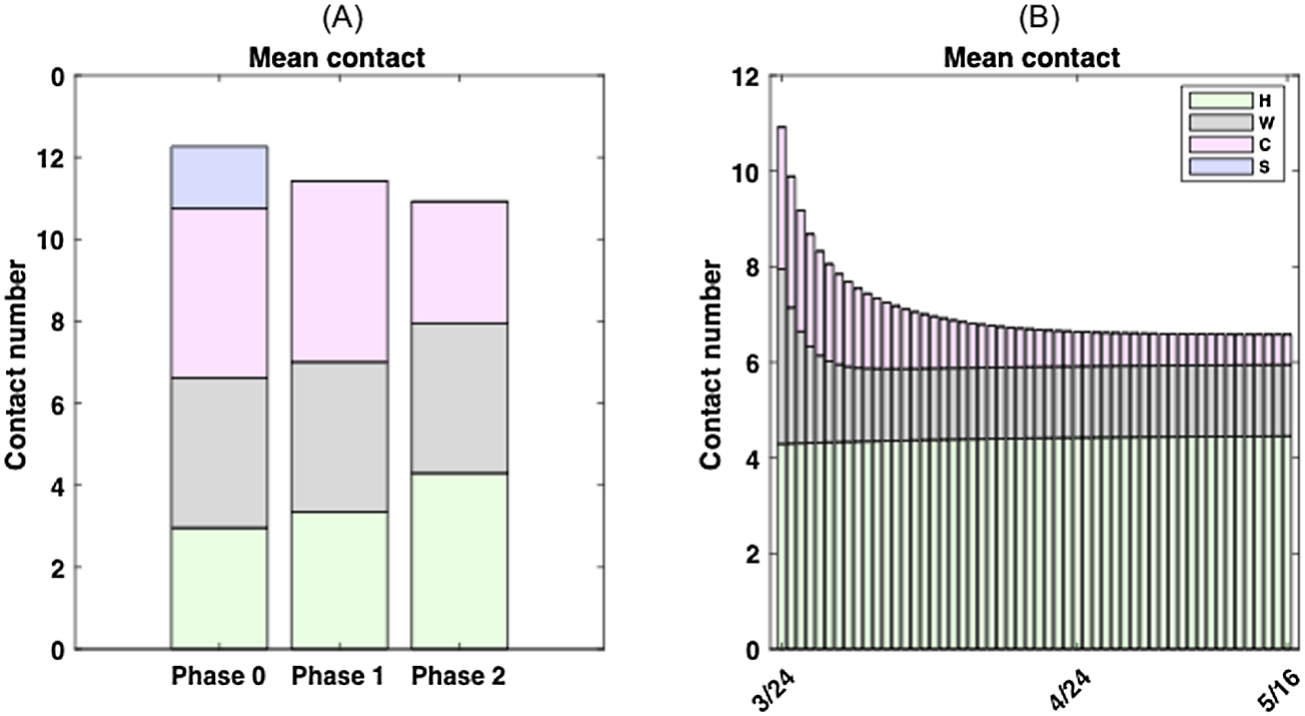
Mean contact rate during four escalation phases of physical distancing measures. We considered four phases of escalation in Ontario: phase 0, monitoring and international travel advisories (until Mar 13); phase 1, public school closure (Mar 14–17); phase 2, physical distancing advisories (Mar 18–23); phase 3, closure of non-essential workplaces (Mar 24–May 16). The contact mixing matrices are constant for phase 0, 1 and 2 and the contact mixing is modelled as time-dependent for phase 3. (**A**) The mean contact rate from phase 0 (12.27), 1 (11.42) to 2 (10.92) including the setting breakdown; (**B**) The time-dependent mean contact rate by setting for phase 3

**Figure 7:**
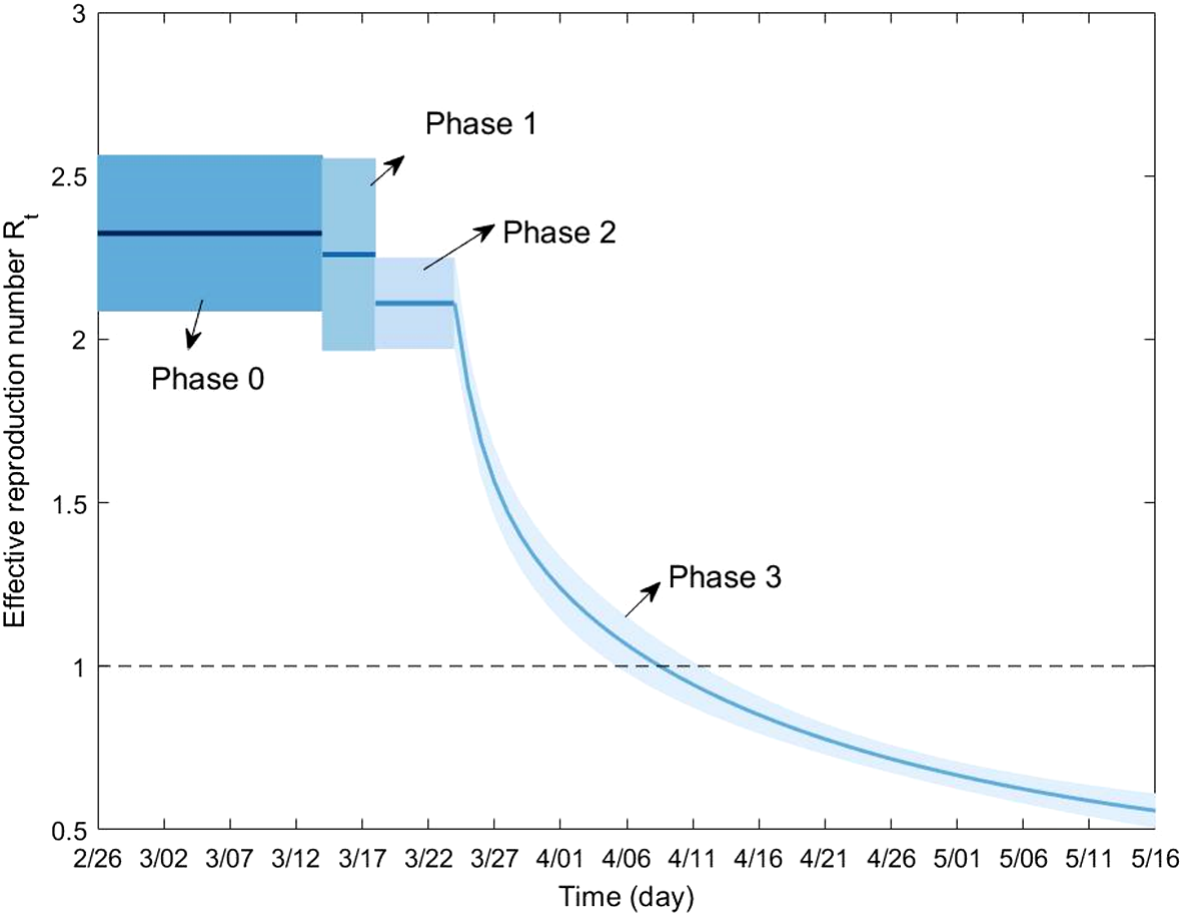
Estimated effective reproduction number $R_{t}$. The solid line represents the estimated mean $R_{t}$ value and the shaded region depicts the 95% confidence interval. $R_{t}$ declines below 1 between April 5 and April 12 following the implementation of a package of non-pharmaceutical interventions

Through model fitting, we estimated the age-independent parameters (Table 2) and age-dependent parameters (Table 4). The model fit with quantified uncertainty against the age-stratified cumulative incidence data is shown in Fig. 3 and with all age classes combined in Fig. 4. By estimating the weights associated with the setting-specific contact matrices, we identified the mixing patterns in all four phases of escalation (Fig. 5) as well as the mean individual contact rate (Fig. 6). The fitted parameters allowed us to estimate the effective reproduction number (Fig. 7) and the mean infectious contact (Table 5), for which we provide comparison with a homogeneous mixing model of similar scope [2].

**Table 5:** Mean infectious contact during different escalation phases

Model	Mean infectious contacts				
	Phase 0	Phase 1	Phase 2	Phase 3 (May 16)	Phase 3 (asymptotic)
Homogeneous model [2]	1.7011	1.4866	1.1825	0.3957	0.3232
Age-structured model	1.4558	1.4386	1.3541	0.6806	0.7555

Calculated from the age-structured model (1) and resulting from the homogeneous modelling analysis in prior modelling work in Ontario [2].

### Setting-specific social contact mixing

**Figure 8:**
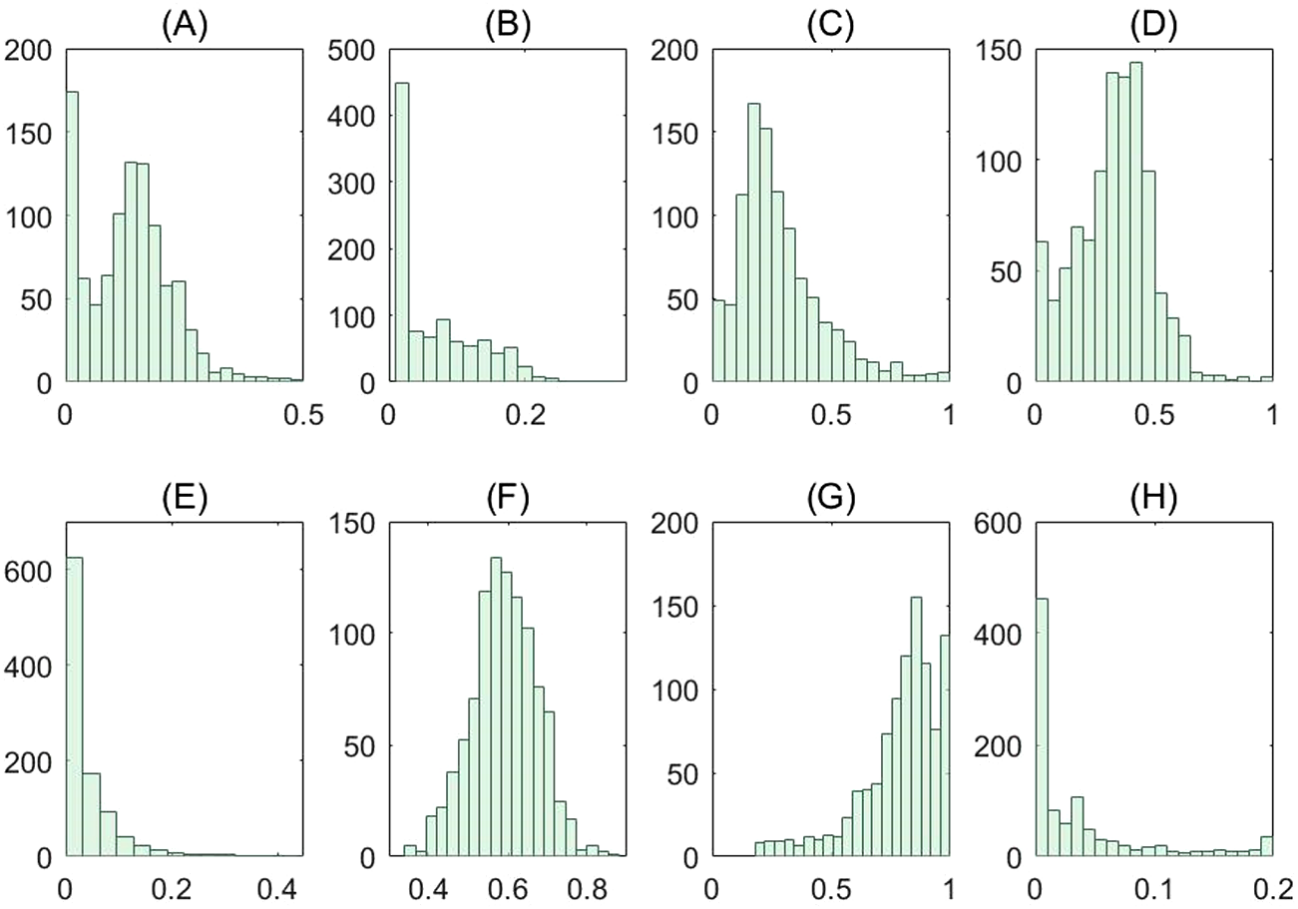
Empirical distributions of model parameters associated with the contact matrices. Empirical distributions of the weights of the contact matrices obtained from the fitting results of the 1000 bootstrap realizations. Panels (A)–(H) correspond to the distributions for the model parameters (**A**) $p_{1}^{H}$, (**B**) $p_{1}^{C}$, (**C**) $p_{2}^{H}$, (**D**) $p_{2}^{C}$, (**E**) $p_{3}^{H}$, (**F**) $p_{3}^{W}$, (**G**) $p_{3}^{C}$ and (**H**) $r_{c}$

We estimated the age-specific contact mixing profile for each phase of escalation (Fig. 5). We estimated the mean contact rate (i.e., the average number of contacts per day of one random individual with the total population) in the absence of physical distancing measures to be 12.27 per day per person in Ontario. This contact rate was assumed during phase 0, which corresponds to the beginning of the epidemic in Ontario where no major physical distancing advisories were in effect (Fig. 6(A)). Figure 6 shows the breakdown of contact rates by their respective setting (school, workplace, household, and general community). Relative to the pre-intervention value, the total increase of household contacts was 13% after school closure, 45% after the additional physical distancing measures, and 51% on May 16 after the closure of non-essential businesses (Table 6). Measures following the closure of non-essential businesses were estimated to have an impact of 59% reduction in the total workplace contacts and 85% community-related contacts as of May 16 (Table 6 and Fig. 6). Table 6 shows a complete summary of the estimated shifts in terms of the mean daily contact rate in Ontario. We also depicted the empirical distributions of the weights of the phase- and setting-specific contact matrices (Fig. 8).

**Table 6:** Estimated mean daily contact rate by setting and escalation phase

Setting	Daily contact rate pre-interventions (contacts/day)	Running daily contact rate (change relative to pre-intervention)		
		Phase 1	Phase 2	Phase 3 (May 16)
School	1.52	0 (−100%)	0 (−100%)	0 (−100%)
Workplace	3.66	3.66 (0%)	3.66 (0%)	1.49 (−59%)
Community	4.14	4.42 (+7%)	2.97 (−28%)	0.64 (−85%)
Household	2.95	3.34 (+13%)	4.29 (+45%)	4.45 (+51%)
Total	12.27	11.42 (−7%)	10.92 (−11%)	6.58 (−46%)

Estimates of the mean individual daily contact rate and its change relative to pre-intervention values in Ontario, Canada.

### Infections acquired in workplace, household, community and school settings

**Figure 9:**
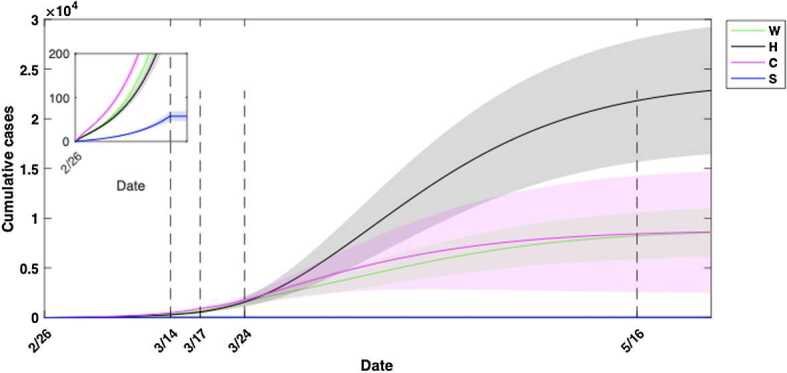
Cumulative infections acquired in workplace, households, community and school settings for all age groups. Community contacts initially contributed to more infections than contacts from remaining three locations, while household contacts played a dominant role in contributing new infections after the closure of non-essential workplaces on March 24. Due to the closure of schools at the beginning of phase 1 on March 14, no more new infections occurred at the school setting (shown in the sub-panel). Estimated mean values are represented by solid lines and the 95% confidence interval (CI) by surrounding shaded regions. CIs based on fitting results to 1000 realizations of the cumulative reported case data in Ontario, Canada

**Figure 10:**
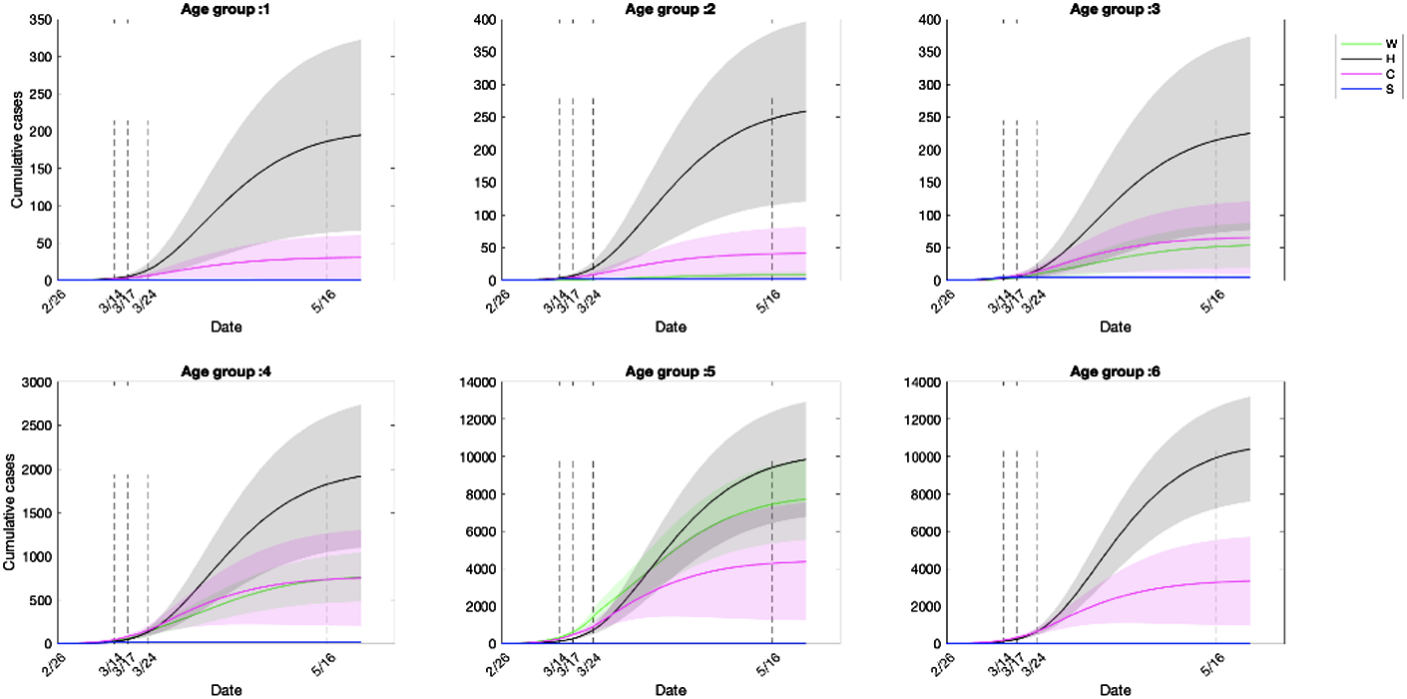
Cumulative infections by age group and setting. Households were the primary location for the estimated transmission for all age groups, while communities or workplaces were the secondary location for different age groups

**Figure 11:**
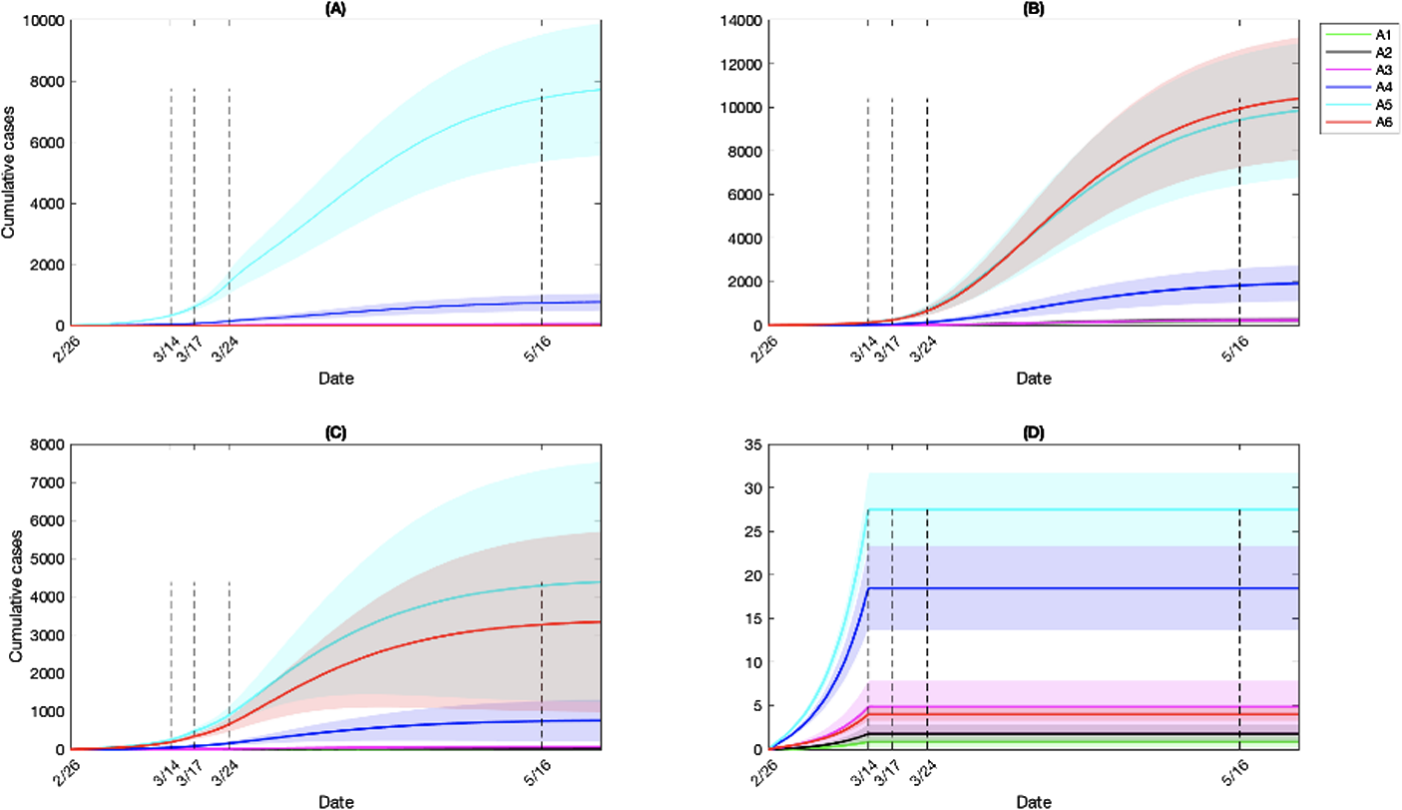
Cumulative infections by setting and age class. Model-estimated cumulative infections acquired in (**A**) Workplaces, (**B**) Household, (**C**) Community and other locations and (**D**) Schools

We quantified the number of cumulative infections acquired in each setting and age group (Fig. 9). During phase 0, the cumulative infections were estimated to primarily result from community contacts, followed by contacts at workplaces and households (Fig. 9). The community contacts played the primary role in contributing effective transmissions till the early stage of phase 3, when the infections from household contacts eventually took over the primary role (Fig. 9). Households are the locations where the highest number of infections were estimated to occur among all age groups by May 16 (Fig. 10). Communities were the second most popular location to gain infections for age groups of individuals younger than 17 and older than 65 (Fig. 10). The age classes composed of young children and individuals aged 65 and above were estimated to gain relatively few infections from workplace contacts, while the working group (ages 25–64) acquired a similar number of infections from workplaces compared to households (Fig. 10). The estimated infections from school setting contacts were relatively few due to the early closure of schools at the beginning of phase 1 (Fig. 10). Overall, the workforce age class (aged 25–64) consistently was estimated to acquire a higher number of infections at workplaces, communities and schools compared to other age groups (Fig. 11). This was followed by individuals aged 18–24 in workplaces and schools (Fig. 11(A)(D)), while households and communities were settings of considerable transmission for the senior age group (aged 65+), shown in Fig. 11(B)(C).

### Age-specific susceptibility to infection and diagnosis probability

**Figure 12:**
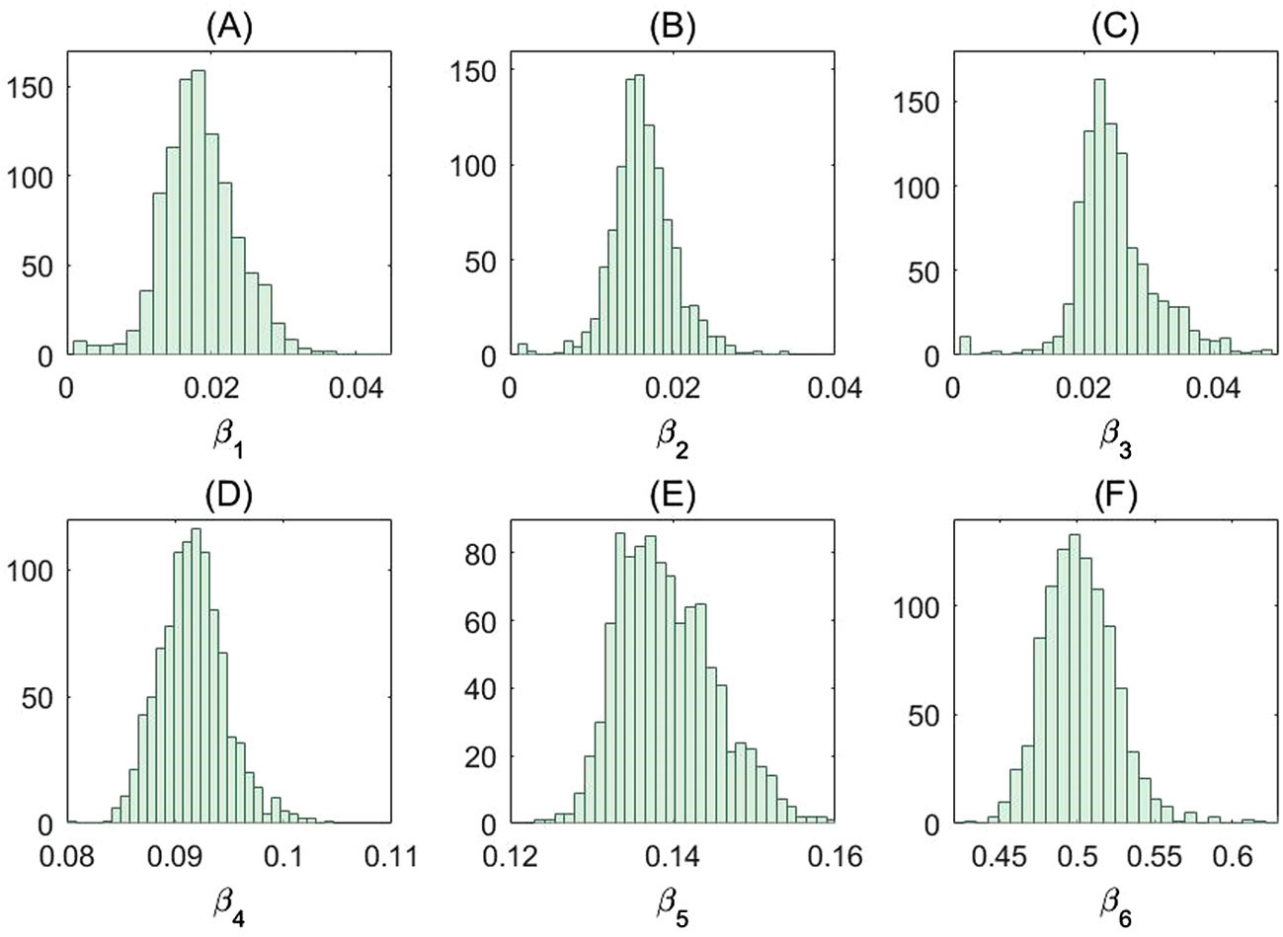
Empirical distributions of the age-specific susceptibility. Estimated parameters obtained from the fitting results of each of the 1000 bootstrap realizations. Empirical distribution of the estimated age-specific susceptibility for ages (**A**) ages 0–5; (**B**) ages 6–13; (**C**) ages 14–17; (**D**) ages 18–24; (**E**) ages 25–64 and (**F**) ages 65+. See Methods for the details

We estimated the age-stratified probability of diagnosis for symptomatic individuals and susceptibility to infection (Table 7). More precisely, the susceptibility to infection in our model refers to the probability of infection upon contact with an infectious individual. We also depicted the empirical distributions of the age-specific susceptibilities corresponding to the uncertainty analysis conducted (Fig. 12). The estimated age-specific susceptibilities are robust to error in the reported case counts, which can be observed from their respective empirical distributions (Fig. 12).

**Table 7:** Age-specific model parameter estimates in Ontario, Canada: Percentage of symptomatic individuals diagnosed, and susceptibility to infection

Parameter	Age class					
	0–5	6–13	14–17	18–24	25–64	65+
Percentage of symptomatic individuals diagnosed	31%	38%	37%	20%	64%	67%
Susceptibility to infection	1.9%	1.6%	2.5%	9.2%	13.9%	50.2%

Percentage of symptomatic diagnosed individuals and susceptibility (i.e., probability of infection upon contact, $\beta _{{i}}$). The reported values are obtained from the mean of the fitting results of 1000 bootstrap realizations. The fraction of symptomatic diagnosed individuals is calculated from $\frac{\delta _{I,i}}{\delta _{I,i} + \gamma _{I}}$, where $\delta _{I,i}$ and $\gamma _{I}$ denote the diagnosis and recovery rate, respectively.

## Discussion

We estimated that the susceptibility to SARS-CoV-2 infection increases with age, which is consistent with findings from prior works [9, 11]. From our estimates, younger age groups (17 and below) have relatively low susceptibility to infection (less than 3%) compared to the senior age class (65+), with a probability of 50.2% to be infected upon contact (Table 7). Further, we estimated the senior class to be the most susceptible to infection (Table 7). This comes in addition to the relatively high vulnerability of the senior age group [10, 15, 23, 24], and provides further support to the necessity of protecting these individuals. The relatively lower susceptibility of younger children suggests that they may not have been a major driver of the COVID-19 epidemic in Ontario until May 16, if their transmissibility was comparable to the remaining age groups, confirming the existing literature [25–27]. We urge caution in interpreting these results, as in light of emerging data, that rapidly increased cases among non-seniors in Ontario indicate that mixing among these age groups and less abidance to physical distancing measures has been evident (for details see Appendix B: Caution in interpretation). Also, our findings of susceptibility are in line with a retrospective cohort observational study conducted in mainland China, which computed a secondary attack rate among household contacts of 12.4–17.1%, with a lower risk of developing the infection among the younger subjects with respect to the elderly [28]. We also estimated an overall increasing trend with age in the probability of diagnosis among symptomatic individuals (Table 7). This finding is both logical and consistent with prior findings [15], as the severity of illness due to COVID-19 has been found to increase with age and cases requiring medical attention may be more likely to be captured by the virologic surveillance system. Finally, our results are in line with the findings of the existing modeling studies [29–32] that have found that a timely and stringent implementation of non-pharmacological interventions are effective in curbing the spread of the ongoing outbreak, if they are enforced until the virus transmission has been significantly reduced.

In this study, we estimated a 46% (12.27 to 6.58) decrease in the mean individual contact rate following the implementation of a series of government interventions in Ontario (Table 6). Studies which also estimated the social contact rates during the COVID-19 era, consisted of contact surveys where participants were asked to provide details about the number and locations of their social contacts. Findings from these studies indicate that physical distancing measures have led to the reduction of daily social contact rates in China [11, 33], Luxembourg [13], and the UK [12], with varying degrees of reduction. The age-stratified contact matrices, in the presence of government interventions, were identified in three studies [11, 12, 33] and their implications explored in terms of impact on the basic reproduction number [12] and model-based analyses [11, 33]. In agreement with our study, the estimated contact mixing patterns following the implementation of interventions had closely resembled the household mixing pattern (Fig. 5) [11, 33]. The estimated average number of daily contacts per participant before and during lockdown, were from 7.9 to 2.2 (72.2% reduction) in Shenzhen, 10.8 to 2.8 (74% reduction) in the UK, 9.5 to 2.2 (76.8% reduction) in Changsha, 18.8 to 2.3 (87.8% reduction) in Shanghai, 17.5 to 3.2 (81.7% reduction) in Luxembourg, to 14.6 to 2 (86.3% reduction) in Wuhan [11–13, 33]. It is possible that the survey-based approaches underestimate the contact rate, as there is a risk of bias from sources such as selection and recall bias. Also, participants may not wish to disclose their true number of contacts during government interventions. These factors may explain differences between the estimates from the two methodologies. Even so, with the data-driven approach presented in this study or the survey-based approach, it has been estimated that a substantial decrease in the individual contact rate occurred following the implementation of government interventions, with a shift to household contact. Overall, through the reduction in social contact rate and alteration of the mixing patterns, model-based analyses indicate interventions have been effective in mitigating transmission [11, 33].

As each physical distancing intervention may cause a shift in contact patterns, for instance by increasing the time that individuals spend at home, estimating the relative increase or decrease of the setting-specific daily contacts in each escalation phase enables the assessment of the expected shift of the infection towards different subpopulations and the contribution of contacts in each setting to the spread of infection. We estimated an effective decrease in contacts in the workplace and community settings; meanwhile, the household contact has increased by 51% from the pre-intervention phase to the end of lockdown phase on May 16 (Table 6). Therefore, additional household transmission should be taken into account by decision-makers when planning and implementing interventions, especially in light of the relatively high contact rates of seniors in household settings.

Estimates of the age- and setting-specific social contact patterns during escalation of physical distancing, together with a deeper understanding of the age-specific susceptibility, allow to investigate different scenarios for reopening the economy, including businesses and schools [30–32]. These estimates provide needed tools to simulate scenarios of staged reopening or reopening targeted to specific subgroups, such as resuming of partial school classes, selected business sectors, etc. Additionally, this framework may be used for scenario analysis such as rotating workforce strategies, where the workforce is divided into groups with different working schedules. The specific choice of the age group subdivision in this study is motivated by age targeted intervention, in the spirit of assessing gradual resumption of schools and workplaces. Further, this framework can be used to incorporate vaccination of different age classes in the event an efficacious vaccine comes to light and identify optimal distribution strategies. Although we have focused primarily on age-stratified contact mixing, susceptibility and symptomatic diagnosis probability in this study, we also used the modelling framework to quantify key control parameters related to the efficacy of contact tracing efforts (for the details, see Appendix C: Control parameter assessment).

This study and its data sources have several limitations. For our analyses, we primarily used cumulative incidence data, which is subject to several forms of error that may result in inaccuracies and biased estimates. Additional sources of error in our study may result from the specific circumstances in Ontario, in which a disproportionate number of health care workers were affected by COVID-19 and outbreaks had occurred in long-term care homes. For a discussion of these details, see Appendix D: Limitations in incidence data.

## Conclusions

The methodology introduced and illustrated in this study aims to provide the much-needed tools for intervention evaluation in terms of inferring the age- and setting-specific contact mixing in rapidly evolving circumstances, without the time and resources required for survey-based approaches. The data-driven, model-based approach can provide insights in almost real time based on incoming data, which is key to inform decision- and policy-making in an emergency situation, such as the current pandemic. We also note that the necessary surveillance data for COVID-19 and demographic data for analyses is readily and publicly available in many regions worldwide. Similarly, the age- and setting-specific mixing matrices utilized within are available in 152 countries [17]. Hence, the methodology can be readily adopted in many regions worldwide and could yield insights of the transmission risk and the effectiveness of different age- and setting (workplace, school, community, and household)-specific interventions.

## Data Availability

The datasets generated during and/or analyzed during the current study are available publicly except for the age-stratified COVID-19 incidence data.

## Appendix A: Baseline contact matrices

To establish a contact matrix representative of contact mixing in Ontario, we utilized the projected Canadian matrices [17] and further adapted the matrices for modelling purposes. Several of the requirements for the target contact matrix to be suitable for integration in the transmission model (1) are:

i. Modified age group subdivisions (6 age groups);
ii. Reciprocity condition is satisfied;
iii. Accounts for the specific age structure of Ontario, Canada, in 2019;
iv. Mean connectivity is representative of individual mean contact rate in Ontario, Canada;
v. Represents contact mixing in distinct social settings (household, workplace, community and other locations, school).

The topics of reciprocity and mean connectivity are discussed further in prior work [34]. Briefly, social contact survey data is subject to over-representation and under-representation of age groups, reporting error, etc.; hence, estimated population-level contacts are generally not symmetric. However, as contacts must be reciprocal, the number of total contacts from age group *i* to *j* must be identical to those contacts from group *j* to *i*. Also, the mean connectivity of each contact matrix, or the average number of contacts per individual in the population, should be preserved during the transformations within the same year. We introduced a series of transformations which address items (i)–(v). We then utilized the resultant contact matrices in the simulations of the transmission model (1) to quantify the age-specific contact mixing in Ontario.

### Method

Denote the reference contact matrices, which are representative of social contact mixing in Canada in year 2006, $C^{H}$, $C^{W}$, $C^{C}$, $C^{S}$ for household, workplace, community and school settings, respectively. The entries of the contact matrix are the number of daily social contacts of a single individual in age class *i* with individuals in age class *j* (units contacts/day as defined by those contacts believed to be relevant for the spread of respiratory illnesses) [16]. We utilized $C^{\mathrm{Ref}}$ to obtain a matrix representative of contact mixing in Ontario, Canada to satisfy the properties (i)–(v) above. The subsequent process is applied separately to each of the setting-specific reference contact matrices.

### Reciprocity correction

We corrected each setting-specific reference matrix $C^{\mathrm{Ref}}$ for reciprocity to ensure that contacts between age classes at the population-level (extensive scale) were reciprocal. Specifically, we ensured symmetry of the population-level contacts and returned back to the individual-level contact scale. Let $E_{ij} = C_{ij}^{\mathrm{Ref}} N_{i}$ represent the extensive scale contact matrix between age class *i* and class *j*, where $N_{i}$ represents the population of age class *i* in Canada in year 2006. To ensure the symmetry of matrix *E*, and thus population-level contacts, we applied the transformation $E_{ij} \rightarrow \frac{1}{2} ( E_{ij} + E_{ij}^{T} )$. We then converted from population-level total contacts to the individual-level contact scale to redefine $C_{ij}^{\mathrm{Ref}}:= \frac{E_{ij}}{N_{i}}$. The resultant $C^{\mathrm{Ref}} $ has now been corrected for reciprocity.

We then adjusted each reference matrix $C^{\mathrm{Ref}}$, for Canada, to the matrix *C*, for Ontario, according to the demography of Ontario using an established method [34]. To accomplish this, we projected the reference contact matrix $C^{\mathrm{Ref}}$ to the target matrix *C* using the transformation

2 $$ C_{ij} = C_{ij}^{\mathrm{Ref}} \frac{N' N_{j} '}{N_{j} \sum_{i,j} C_{ij}^{\mathrm{Ref}} \frac{N_{i} ' N_{j} '}{N_{j}}}, $$

where $N_{j}$ ($N_{i} $) and $N_{j} '$ ($N_{i} ' $) are the number of individuals in age class *j* (*i*) in Canada and Ontario in 2006, respectively. Also, *N* and $N'$ are the total numbers of individuals in the reference year Canada and Ontario in 2006, respectively. Equation (2) may be interpreted as an adjustment of the contact rate based on the ratio of the target population density of available contactees in Ontario and density of contactees in the reference setting in Canada. Since this transformation adjusted the matrix from the country setting to a provincial setting within the same year, we also normalize the matrix to have a mean degree (or mean connectivity) of 1 during the transformation in order to preserve the mean connectivity. After the transformation, we rescale the matrix *C* to the original degree of $C^{\mathrm{Ref}}$ by multiplying the mean connectivity

$$ \langle k\rangle = \frac{1}{N} \sum_{i,j} C_{ij}^{\mathrm{Ref}} N_{i}. $$

We preserve the mean connectivity in this transformation, i.e., the average number of daily contacts per individual in the population is assumed to be equal in Ontario and Canada in the same year. We note that an alternative density transformation could also be used, which relaxes this assumption and allows the mean connectivity to depart from its original value (for instance, method M2 in [34]). To quantify the setting-specific contact mixing, we applied this above process, using Equation (2), to the established household, workplace, community, and school contact matrices for Canada [17].

### Method to age-transform contact matrix

We outline the process used for generating contact matrices in the desired age subdivision format by utilizing known contact mixing data and Canadian demographic data. The key concept was to utilize established contact data which informed a $16 \times 16$ contact matrix for age groups 0–4, 5–9, 10–14, 15–19, 20–24, 25–29, 30–34, 35–39, 40–44, 45–49, 50–54, 55–59, 60–64, 65–69, 70–74, 75+, and use a series of property-preserving transformations to generate a desired or target $6 \times 6$ contact matrix for age groups 0–5, 6–13, 14–17, 18–24, 25–64, 65+. Here, we conducted the transformation between different age groups for the year 2006. Based on the homogeneous mixing assumption and a conservation law on contacts received and offered in each age group, we calculated the entries of the target $6 \times 6$ matrix using the following formula

$$ C_{kl}^{6} = \frac{1}{N_{k}^{6}} \sum _{i=1}^{16} \sum_{j=1}^{16} C_{ij}^{16} N_{i} \frac{\overline{N}_{jl}}{N_{j}} \frac{\overline{N}_{ik}}{N_{i}}, $$

where $\overline{N}_{jl}$ ($\overline{N}_{ik}$) represents the overlapped population in the old age group *j* (*i*) and new age group *l* (*k*). And $C_{ij}^{16}$ and $C_{kl}^{6}$ are the entries of the contact matrix for the old and new age structure, respectively. $N_{k}^{6}$ is the population in age group *k* for the new age structure. This transformation preserves the mean connectivity and reciprocity from the previous transformation.

### Method to project contact matrix to different years

We now have obtained the contact matrix among 6 age groups in 2006, and the following transformation projects the contact matrix to 2019 based on the Canadian demographic data. In what follows, $N_{j}$ and $N_{j} '$ are the number of individuals in the *j*th age group in the original (i.e., 2006) and projected (i.e., 2019) year, *N* and $N '$ are the total population in the original and projected year. Then, we have

$$ C_{ij}^{\mathrm{projection}} = C_{ij}^{\mathrm{original}} \frac{N N_{j} '}{N_{j} N '}. $$

The contactee correction terms of the form $\frac{N N_{j} '}{N_{j} N '}$ represents the ratio of contactees in the projected year to those in the origin year. With the contactee density correction terms, we expressed all entries of the projected contact matrix in terms of the known, original contact matrix entries. Equations were kept general to hold true when *N* may differ from $N'$. The above equation may also be interpreted as an adjustment of the contact rate based on the ratio of projected population density of contactees and density of contactees in the original setting. Due to the variations in population profile in different timing, the average connectivity is not preserved exactly; however is representative of mean contact rate in Ontario. We note that an alternative series of transformations could be utilized to preserve the mean connectivity of the Canadian reference contact matrix. Finally, this transformation preserves the reciprocity.

### Results

The contact matrices resulting from the series of transformations outlined are shown in Fig. 2 and are representative of mixing in Ontario. We then utilized the resultant contact matrices $C^{H}$, $C^{W}$, $C^{C}$, $C^{S}$ in the simulation of transmission model (1) to quantify heterogeneity in age-specific and setting-specific contact mixing in Ontario.

## Appendix B: Caution in interpretation

The predicted trajectory as of May 16 largely underestimated the cases reported in Ontario as of mid-July (Fig. 3). However, observing the age-stratified model fit vs. cumulative incidence data (Fig. 4), we see that the disparity is among those aged less than 65. The underestimation may be due to the relaxation of measures starting from May 16 and/or decreased abidance to physical distancing measures. For instance, possible culprits include increased warm weather in Ontario and a national holiday weekend. While our study suggests that younger individuals are less susceptible; the observed cases following relaxation suggests increased transmission among these groups may be due to increased mixing among those non-senior individuals.

## Appendix C: Control parameter assessment

The transmission model accounts for two types of detection routes: contact tracing and the diagnosis of individuals presenting symptoms. Estimates of the parameters related to these processes can be utilized to assess the efficacy of interventions implemented and conduct modelling scenario analyses. Our estimates show that the fraction of infectious contacts that were effectively traced and isolated increased from 12% before phase 3 to a limit value of 73% during phase 3 (Table 2). Parameterizing the transmission model with region- or country-specific demographic and incidence data could also allow a comparative evaluation of interventions between different regions. Furthermore, the evaluation of the current levels of contact tracing and diagnosis efforts is fundamental for planning public health policies [2, 3].

## Appendix D: Limitations in incidence data

For our analysis we used cumulative incidence data, which is subject to several forms of error, including underreporting (COVID-19 positive individuals not having their illness reported) and under-ascertainment (cases not seeking health care), as not all COVID-19 cases are captured by the surveillance system. We note that there may be heterogeneity by age in the proportion of individuals seeking health care and testing due to illness, hence the estimated age-specific parameters are impacted by age-specific reporting rates and ascertainment rates. Specifically, the severity of illness due to COVID-19 has been found to increase with age [15] and cases requiring medical attention may be more likely to be captured by the surveillance system, which is consistent with our findings of diagnosis rate increasing with age (Table 7). However, we stress that testing protocols in Ontario have been variable during the course of the epidemic, altering the scope of individuals and symptoms that have been tested for COVID-19, hence making it difficult to obtain robust estimates when those rates are assumed constant over time. Analyzing data while interventions are in place introduces natural biases: the low relative susceptibility in younger age groups may be a result of early school closure, which prevented transmission in younger age groups very early on in the Ontario epidemic. Such an effective containment measure may not have been possible in settings such as Ontario’s long-term care homes where the prominent age class is seniors and transmission continued.

Additional sources of error in our study result from the specific situation in Ontario. As of June 3, 2020, Greater Toronto Area public health units accounted for 66.4% of cases. It may then be more appropriate to consider modelling in smaller regions or public health units as cases are not evenly dispersed throughout the province. In addition, as of June 3, 2020, a proportion of 17.7% and 6.3% of all cases were among long-term care home residents and among health care workers associated with long-term care outbreaks, respectively. Heterogeneity in geographical location or finer granularity to the level of long-term care homes and hospitals are not captured by the current model.
